# Active MMP‐8 point‐of‐care (PoC)/chairside enzyme‐test as an adjunctive tool for early and real‐time diagnosis of peri‐implantitis

**DOI:** 10.1002/cre2.537

**Published:** 2022-02-03

**Authors:** Hanna Lähteenmäki, Taina Tervahartiala, Ismo T. Räisänen, Pirjo Pärnänen, Matti Mauramo, Shipra Gupta, Victoria Sampson, Nilminie Rathnayake, Anna‐Maria Heikkinen, Saeed Alassiri, Dirk‐Rolf Gieselmann, Roland Frankenberger, Timo Sorsa

**Affiliations:** ^1^ Department of Oral and Maxillofacial Diseases University of Helsinki and Helsinki University Hospital Helsinki Finland; ^2^ Department of Pathology, Haartman Institute and HUSLab University of Helsinki and Helsinki University Hospital Helsinki Finland; ^3^ Unit of Periodontics, Oral Health Sciences Centre, Post Graduate Institute of Medical Education & Research (PGIMER) Chandigarh India; ^4^ Department of Periodontics and Community Dental Sciences King Khalid University Abha Saudi Arabia; ^5^ Institute for Molecular Diagnostics IMOD Solingen Germany; ^6^ Department for Operative Dentistry, Endodontics, and Pediatric Dentistry Philipps University Marburg and University Hospital Giessen and Marburg Marburg Germany; ^7^ Division of Periodontology, Department of Dental Medicine Karolinska Institutet Huddinge Sweden

**Keywords:** biomarkers, diagnosis, matrix metalloproteinase 8, peri‐implantitis, preventive medicine

## Abstract

**Objective:**

The aim of this study was to investigate the utility of the active matrix metalloproteinase (aMMP‐8)‐point‐of‐care (PoC) test as a quantitative real‐time chair‐side diagnostic tool for peri‐implant diagnosis, as well as assess the potentially developing and ongoing risk relative to the traditional clinical methods.

**Background:**

Current peri‐implant and periodontal disease diagnoses rely on clinical and radiological examinations. This case‐control study investigated the applicability of aMMP‐8‐PoC immunotest for quantitative real‐time diagnosis and monitoring of dental implants in health and disease.

**Methods:**

Sixty‐eight patients visiting a specialist clinic for maintenance following dental implant placement underwent assessment of their peri‐implant health. aMMP‐8‐PoC peri‐implant sulcular fluid (PISF) lateral‐flow immunotests were performed using ImplantSafe® technology quantitated by ORALyzer®. In addition, the PISF samples were analyzed for total MMP‐8, calprotectin, and interleukin (IL)‐6 by enzyme‐linked immunosorbent assays (ELISA), aMMP‐8 by western immunoblot, and MMP‐2 and MMP‐9 by gelatin zymography.

**Results:**

The aMMP‐8‐PoC test promptly recorded and reflected peri‐implant disease, differentiating it clearly from health. X‐ray findings (bone loss > 2 mm), peri‐implant pocket depth ≥ 3 mm, and bleeding on probing were significantly more prevalent among implants positive for the aMMP‐8‐PoC test. aMMP‐8/ORALyzer analysis was more precise in recording disease than total MMP‐8, calprotectin, IL‐6, MMP‐2, and MMP‐9.

**Conclusions:**

The aMMP‐8‐PoC test can be conveniently implemented to alert for and detect active collagenolysis affecting peri‐implant tissues, both in the early and advanced stages of the disease. Active and fragmented MMP‐8 exhibits a strong and significant association with peri‐implantitis as compared to total MMP‐8 and other biomarkers and can be utilized as the POC/chairside biomarker of choice in the new classification of peri‐implantitis.

## 1 INTRODUCTION

The popularity of dental implants as a treatment option for replacing missing teeth has substantially increased over the past 20 years (Elani et al., 2018). This development poses a challenge to oral health professionals because although in many cases dental implant treatments have been successful having a high survival rate, implant complications and failures have been frequent (Pjetursson et al., 2012). Peri‐implantitis, a pathological inflammatory condition around dental implants, is a major risk factor for late dental implant failures (Manor et al., 2009; Schwarz et al., 2018). Previous studies have estimated that the prevalence of peri‐implantitis may be as high as 23% of dental implants, while peri‐implant mucositis, which is considered a precursor for peri‐implantitis, may affect more than 40% of implants (Derks & Tomasi, 2015; Heitz‐Mayfield & Salvi, 2018).

Current peri‐implant and periodontal disease diagnosis rely on clinical and radiological examinations, which have been commonly used by all oral health professionals for decades and are easy and practical to interpret. However, clinicians are only able to detect and measure peri‐implant/periodontal diseases after clinical manifestations have occurred using the traditional methods. To overcome this limitation, biomarkers have been studied extensively (Alassy et al., 2019; Arias‐Bujanda et al., 2019; Carinci et al., 2019; Gul et al., 2020). Key biomarkers of peri‐implant and periodontal tissue destruction, once identified, could alert the clinician as to the onset of collagen breakdown, long before clinical manifestations set in. In adjunct with the traditional methods, they could increase the accuracy of early detection of peri‐implant and periodontal diseases, prediction of disease progression, and monitoring of treatment effects (Gul et al., 2020).

Periodontal and peri‐implant connective tissue consists mainly of Type I collagen. Results from previous studies support the concept of matrix metalloproteinase‐8 (MMP‐8), also known as neutrophil collagenase or collagenase‐2, as a potential key biomarker responsible for connective tissue destruction or active degeneration of periodontal and peri‐implant disease (Alassy et al., 2019; Al‐Majid et al., 2018; Arias‐Bujanda et al., 2019; Carinci et al., 2019; Gul et al., 2020; Sorsa et al., 2016). MMP‐8 is a major host‐derived collagenolytic proteinase and is regarded as primarily responsible for the irreversible destruction of periodontal and peri‐implant tissues (Buduneli, 2020; Gul et al., 2020; Sorsa et al., 2006). MMP‐8 is responsible for the disintegration and processing of collagens and bioactive inflammatory nonmatrix mediators, not only in various inflammatory and malignant tissue destructive diseases but also in wound healing, immune response and tissue remodeling (Buduneli, 2020; Dejonckheere et al., 2011). It can break down almost all the proteinaceous structural components of connective tissues and basement membranes, as well as process distinct bioactive nonmatrix inflammatory immune mediators. It can also act in a degradative manner upon serpins and insulin receptors (Lauhio et al., 2016; Sorsa et al., 1993).

MMPs, such as MMP‐8, are secreted from the cell as latent pro‐MMPs (Nagase, 1997). The presence of a proteinase susceptible “bait” region in the propeptide allows reactive oxygen species, tissue and plasma proteinases, or opportunistic microbial proteinases (alone or in concert) to activate pro‐MMPs. Once activated, the catalytically competent MMP, such as active MMP‐8 (aMMP‐8), acts as a potential initiator of interstitial collagenolysis at inflammatory sites. It is the pathologically elevated concentration of active MMP‐8 and not the total or latent form, which has been demonstrated to distinguish healthy tissue from gingivitis, periodontitis, peri‐implant mucositis, pre‐implantitis, and peri‐implantitis (Kiili et al., 2002; Lähteenmäki et al., 2020; Lee et al., 1995; Ma et al., 2000; Mancini et al., 1999; Romanelli et al., 1999; Romero‐Castro et al., 2020; Sorsa, Alassiri, et al., 2020; Sorsa, Bacigalupo, et al., 2020; Sorsa et al., 2006, 2010, 2016; Verhulst et al., 2019; Wang et al., 2016; Wohlfahrt et al., 2014). In healthy periodontal and peri‐implant tissues, the concentration of the active form of MMP‐8 is significantly lower or absent altogether, indicating a healthy status, compared to more severe inflammatory diseased conditions in these tissues (Gangbar et al., 1990; Golub et al., 2020; Keles Yucel et al., 2020; Kivelä‐Rajamäki et al., 2003; Lee et al., 1995; Mancini et al., 1999; Räisänen et al., 2018; Romanelli et al., 1999; Sorsa, Bacigalupo, et al., 2020; Sorsa et al., 2006; Teronen et al., 1997; Xu et al., 2008). The aMMP‐8 enzyme, unlike total MMP‐8, shows consistent and sustained pathological elevation that can be assessed from the pocket/peri‐implant pocket fluid and mouthwash by aMMP‐8 analysis (Alassiri et al., 2018; Gangbar et al., 1990; Golub et al., 2020; Izadi Borujeni et al., 2015; Lee et al., 1995; Lorenz et al., 2017; Mancini et al., 1999; Öztürk et al., 2021; Romanelli et al., 1999; Schmalz et al., 2019; Sorsa, Alassiri, et al., 2020).

The aim of this study was to investigate the utility of the aMMP‐8‐PoC/chairside enzyme (ImplantSafe/ORALyzer®)‐test as a quantitative real‐time chair‐side diagnostic tool for peri‐implant diagnosis, as well as assess the potentially developing and ongoing risk relative to the traditional clinical methods. Furthermore, the diseased and healthy peri‐implant sulcular fluid (PISF) samples were analyzed by independent immunoassays, for total MMP‐8, calprotectin, and interleukin (IL)‐6 by enzyme‐linked immunosorbent assays (ELISA) analysis and additionally by western immunoblotting for MMP‐8, and MMP‐2 and MMP‐9 were evaluated by utilizing gelatin‐zymography to compare them with the aMMP‐8‐PoC/chairside test.

## 2 MATERIALS AND METHODS

### 2.1 Study population

Following written informed consent, 68 patients visiting a private clinic “Hammasklinikka Kruunu” in Tampere, Finland, for dental implants were randomly selected for this case‐control study. This study received approval from the local ethical committee of the Helsinki University Hospital, Finland (106§/26.06.2019; dnro HUS/1271/2019) and Regionala etikprövingsnämnden i Stockholm, (EPN) (2016‐08‐24/2016/1:8 and 2016‐1‐24; Dnr 2016/1410‐31/1) in accordance with the Helsinki Declaration. The study was performed from July 2019 to January 2020. Patients who were males or females, smokers or nonsmokers, at least 45 years of age, and who had one or more dental implants were included in this study. If the patient had more than one dental implant, only one implant was randomly chosen and an aMMP‐8 PoC test was performed in the buccal sulcus of that implant. Patients who had used anti‐inflammatory medication and/or antibiotics or had received peri‐implant treatment within the last 6 months were excluded from this study. Dental implants (Nobel Biocare® implants) had been surgically placed according to routine surgical procedures by an experienced dental implant specialist and maintenance therapy was advised at a gap of 6–12 months following implant placement. The surface types of dental implants placed varied between crowns and bridge structures. At the maintenance visit, oral fluid samples were collected and oral examination including bleeding on probing (BOP) was performed and pocket depths at six sites per implant (disto‐buccal, mid‐buccal, mesio‐buccal, disto‐palatal, mid‐palatal, mesio‐palatal) were recorded using a standard millimeter probe (Hu‐Friedy Manufacturing Co., LLC). An X‐ray of the implant area was also taken to assess alveolar bone destruction. Finally, maintenance therapy was provided at the end of the visit following the Swedish National Guidelines (https://www.socialstyrelsen.se/globalassets/sharepoint-dokument/artikelkatalog/nationella-riktlinjer/2011-5-1.pdf). The treatment effect of anti‐infective treatment on peri‐implantitis was measured after 4–6 weeks after baseline by the aMMP‐8 PoC test.

Previous studies demonstrate a large effect size for site‐specific aMMP‐8 measurements by immunofluorometric assay (IFMA) and POCT utilizing the same monoclonal antibodies in peri‐implantitis diagnostics (Alassiri et al., 2018; Golub et al., 2020;  Lähteenmäki et al., 2020; Rathnayake et al., 2017; Sorsa, Bacigalupo, et al., 2020). Based on these studies, a minimum of 26 participants per group was calculated to identify the differences between healthy and peri‐implantitis groups with α = .05 and power = 0.80. Two groups were enrolled: subjects with peri‐implantitis (*n* = 26) and healthy subjects (*n* = 42). Peri‐implantitis was defined as having the combination of X‐ray findings (bone loss > 2 mm), BOP, and peri‐implant pockets of ≥3 mm around the dental implant, while healthy controls were defined as the absence of these three clinical measurements and parameters. Clinical peri‐implant measurements were performed by a trained and calibrated dental hygienist (H. L.).

### 2.2 Analyses of PISF samples

#### 2.2.1 aMMP‐8 analysis using ImplantSafe test kit and ORALyzer

aMMP‐8‐PoC lateral flow immunoassay test, ImplantSafe® Kit (Dentognostics) was performed by a trained dental hygienist before the maintenance therapy was initiated, in accordance with the manufacturer's instructions, as described by Golub et al. (2020), Lähteenmäki et al. (2020), Sorsa, Bacigalupo, et al. (2020). Briefly, the PISF strip of the test kit was placed apically into the peri‐implant sulcus for 30 s, after which the strip was placed in the vial containing 0.6 ml of elution buffer for 5 min. Afterward, a dipstick from the test kit was dipped into the elution fluid for 15 s and then removed, ready for analysis with the ORALyzer® reader (Dentognostics GmbH). Five minutes later, the quantitative result was noted from the result window of the reader. The qualitative result was visible as blue lines on the dipstick; a single blue line indicating an aMMP‐8 level less than 20 ng/ml (negative); and two blue lines as aMMP‐8 level more than 20 ng/ml (positive). The visible result on the dipstick was documented with a photograph too. Among the positive cases with two lines there existed both weak or thin(ner) (a weak positive) or strong or thick(er) (a strong positive) second line (Sorsa et al., 2021). The remaining elution fluid of PISF was used for further analysis in the study and was analyzed for MMP‐2 and MMP‐9 as well as total MMP‐8, IL‐6, and calprotectin by Western immunoblotting, gelatin‐zymography, and ELISA as described in Sections 2.2.2, 2.2.3, 2.2.4.

#### 2.2.2 Western immunoblotting for MMP‐8

The molecular forms of MMP‐8 were detected by a modified enhanced chemiluminescence Western blot analysis kit according to the protocol recommended by the manufacturer (GE Healthcare) and described by Gürsoy et al. (2018) and Hanemaaijer et al. (1997). Briefly, the PISF samples were mixed with Laemmli sample buffer without any reducing reagents and heated for 5 min, followed by protein separation with 11% sodium dodecyl sulfate (SDS)‐polyacrylamide gels with prestained low range molecular weight sodium dodecyl sulfate–polyacrylamide gel electrophoresis (SDS‐PAGE) standards (Bio‐Rad) as molecular weight marker and human neutrophil (PMN) MMP‐8 (ProteaImmun) as a positive control. Target detections were performed by using a primary antibody, polyclonal anti‐MMP‐8, and a horseradish peroxidase‐linked secondary antibody (GE Healthcare). The immunoblots were quantified by GS‐700 Imaging Densitometer Scanner (Bio‐Rad) using the Quantity One program (Bio‐Rad).

#### 2.2.3 Gelatinolytic activity assay for MMP‐2 and MMP‐9

The gelatinolytic activity was assayed from PISF samples with zymographic technique, using 11% SDS‐polyacrylamide gels impregnated with 1 mg/ml gelatin (Merck) as substrate, in line with previous descriptions by Paju et al. (2001) Sorsa et al.  (1997). Before the electrophoresis, the samples were incubated with Laemmli sample buffer without any reducing reagents for 2 h at room temperature. Prestained low range molecular weight SDS‐PAGE standards (Bio‐Rad) were used as molecular weight markers and human MMP‐9 (Merck) as a positive control. After electrophoresis, the gels were washed two times with 50 mM Tris‐HCl buffer and then incubated in 50 mM Tris‐HCl buffer, pH 7.5, containing 0.02% NaN3, 0.5 mM CaCl_2_, and 1 μM ZnCl2 overnight at 370 C. The gelatinolytic activity was then visualized with 1% Coomassie Brilliant Blue R 250 solution; clear bands against the blue background on stained gels. The intensities of gelatinolytic activities were evaluated with GS‐700 Imaging Densitometer Scanner using Quantity One‐program (Bio‐Rad).

#### 2.2.4 ELISA for total MMP‐8, calprotectin, and IL‐6

Total MMP‐8, IL‐6, and calprotectin were measured in the collected PISF samples using ELISA (Quantikine ELISAs, R&D systems). ELISAs were performed according to the manufacturer's protocols. The detection limits of R&D systems Quantikine kits for total MMP‐8, calprotectin, and IL‐6 were 0.013, 0.086, and 0.70 pg/ml, respectively (Lähteenmäki et al., 2020).

### 2.3 Statistical analysis

General patient characteristics were summarized for study patients, those with dental peri‐implantitis, and healthy controls. The differences between peri‐implantitis and healthy controls were compared by Fisher's exact test, Mann–Whitney *U* test, or *t* test. Levels of biomarkers (aMMP‐8, total MMP‐8, calprotectin, and IL‐6) in peri‐implantitis and healthy groups were compared with Mann–Whitney *U* test and *t‐test* (unadjusted model) and logistic regression (adjusted model: biomarker, gender, age of dental implant and smoking). Based on the test of normality (Shapiro–Wilk) Mann–Whitney *U* test (non‐normality) or *t‐test* (normality) was used.

Diagnostic accuracy of the biomarker candidates to discriminate peri‐implantitis from a healthy implant was studied by receiver operating characteristic (ROC) analysis and the area under the ROC curve (AUC). This was carried out using each biomarker and its adjusted logistic regression model (adjusted for gender, age of dental implant, and smoking). The Youden index was used to define the optimal cut‐offs from the ROC curves for each biomarker (unadjusted and adjusted models). Diagnostic sensitivity (Se), specificity (Sp), the percentage of false negatives (FN) and false positives (FP), test accuracy (Acc), and Matthews correlation coefficient (MCC) were calculated for each biomarker by using the cut‐off to assess the quality of classification.

There were two patients with peri‐implantitis and four with a healthy implant that had missing values in the age of dental implant. Thus, the total sample size for unadjusted calculations was 68, and that for adjusted calculations, 62.

All statistical calculations were performed and figures were created using SPSS Statistics, version 27 (IBM Corp.), and JMP® Pro, version 15 (SAS Institute Inc.). Statistical significance was set at 0.05 (two‐tailed).

## 3 RESULTS

### 3.1 Patient characteristics

Sixty‐eight adults with dental implants were assessed. There were 26 males and 42 females, and their ages ranged from 51 to 92 years. No significant differences were found between peri‐implantitis and healthy groups in patients’ age, gender, smoking habits, or systemic diseases. Age of dental implant was significantly higher (mean difference 1.46 years) in the peri‐implantitis group (*p* = .030). The demographic characteristics of the peri‐implantitis patients and their healthy controls are shown in Table 1.

**Table 1:** General characteristics of the patients by group (*n* = 68)

Characteristic	Peri‐implantitis (*n* = 26)	Healthy (*n* = 42)	*p* value^a^
Gender			
Male	11	15	.616
Female	15	27	
Patient's age (years)			
Mean ± SD	68.77 ± 9.89	71.45 ± 7.67	.215
Min–max	51–89	58–92	
Age of dental implant (years)^b^			
Mean ± SD	7.46 ± 3.06	6.00 ± 5.05	.030
Min–max	3–12	0–20	
Smoking			
Yes	7	8	.550
No	19	34	
Diabetes			
Yes	0	1	1.000
No	26	41	
Asthma			
Yes	2	5	.700
No	24	37	
Rheumatic			
Yes	2	4	1.000
No	24	38	
Heart disease			
Yes	9	18	.612
No	17	24	

Abbreviations: BOP+, bleeding on probing; BOP‐, no bleeding on probing; SD, standard deviation.

^a^ Fisher's exact test was used for gender, smoking, diabetes, asthma, rheumatic, and heart disease; *t test* was used for Patient's age and Mann–Whitney *U* test for Age of dental implant.

^b^ Two peri‐implantitis and four healthy patients with missing information about the age of dental implant.

### 3.2 Biomarker levels in PISF and peri‐implant health

**Figure 1:**
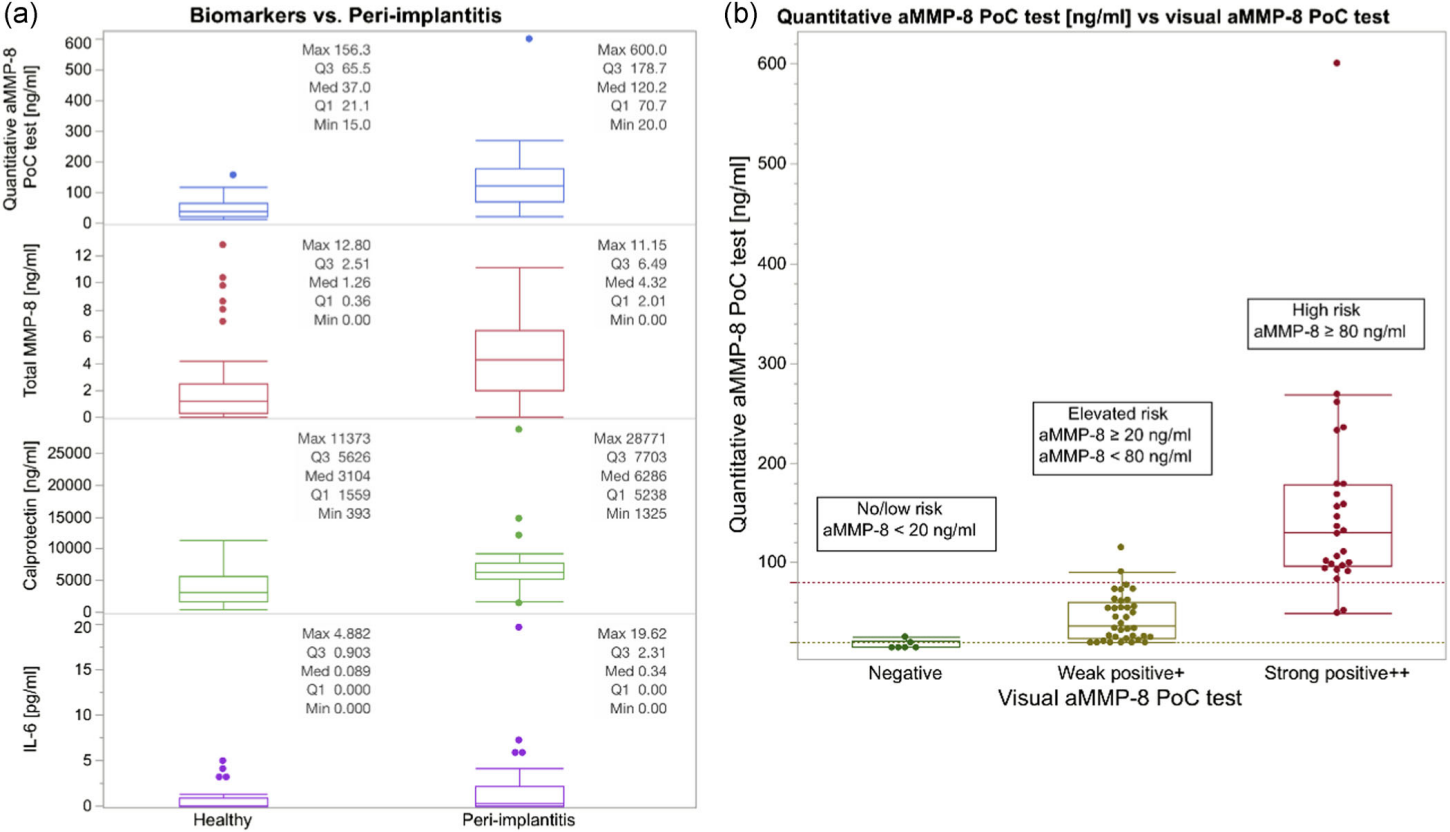
(a) Boxplots of biomarker concentrations per healthy implant and peri‐implantitis groups; (b) quantitative versus visual active matrix metalloproteinase (aMMP)‐8 point‐of‐care test and estimates of cut‐offs for peri‐implant health and peri‐implantitis maximizing the agreement between the two aMMP‐8 tests (*n* = 68)

Analysis of biomarker levels in PISF was performed for aMMP‐8, total MMP‐8, calprotectin, and IL‐6 to assess their potential association with peri‐implantitis (Table 2). Levels of all biomarkers were higher in dental implants with peri‐implantitis than in healthy controls (Figure 1a). Both quantitative and visual aMMP‐8‐PoC tests were positively associated with peri‐implantitis (*p* < .001), even when adjusted for gender, age of dental implant, and smoking in a logistic regression model (*p* < .001). Similarly, total MMP‐8 and calprotectin showed a positive association with peri‐implantitis when unadjusted (*p* < .001 and *p* < .001, respectively) and adjusted (*p* = .005 and *p* = .004, respectively). The association with peri‐implantitis was significant for IL‐6 only in the adjusted model (*p* = .044).

**Table 2:** Levels of biomarkers among healthy dental implants or with peri‐implantitis (*n* = 68)

Characteristic	Peri‐implantitis (*n* = 26)	Healthy (*n* = 42)	Unadjusted *p* value^a^	Adjusted *p* value^b^
Quantitative aMMP‐8 PoC test (ng/ml)	142.32 ± 117.52	49.25 ± 33.45	<.001	<.001
Visual aMMP‐8 PoC test				
Negative −	0	6		
Weak positive +	8	28	<.001	<.001
Strong positive ++	18	8	<.001	<.001
Total MMP‐8 (ng/ml)	4.62 ± 3.16	2.33 ± 3.17	<.001	.005
Calprotectin (ng/ml)	7306.46 ± 5241.21	3999.62 ± 3149.57	<.001	.004
IL‐6 (pg/ml)	2.14 ± 4.13	0.66 ± 1.15	.052	.044

Abbreviations: aMMP, active matrix metalloproteinase; IL, interleukin; PoC, point‐of‐care.

^a^ Mann–Whitney *U* test and *t* test based on test of normality (Shapiro–Wilk).

^b^ Logistic regression model (biomarker test for aMMP‐8, total MMP‐8, calprotectin or IL6 adjusted for gender, age of dental implant, and smoking.

Figure 1b illustrates the distribution of quantitative aMMP‐8‐PoC test results (aMMP‐8 concentrations) in relation to visual aMMP‐8‐PoC test results (negative test, and weak/strong positive test). In that regard, the significant positive association between visual aMMP‐8‐PoC test and peri‐implantitis (*p* < .001; Table 2) already showed that prevalence of peri‐implantitis increased as negative test results changed to weak, and strong positives, that is, aMMP‐8 concentration increased. The largest agreement between the quantitative and visual aMMP‐8‐PoC tests was obtained by setting two thresholds: aMMP‐8 = 20 and 80 ng/ml (Figure 1b). They can be used as estimates of cut‐offs for peri‐implant health and peri‐implantitis risk: no/low risk (aMMP‐8 < 20 ng/ml), elevated risk (aMMP‐8 ≥ 20 and aMMP‐8 < 80 ng/ml), and high risk (aMMP‐8 ≥ 80 ng/ml) (Figure 1b).

**Figure 2:**
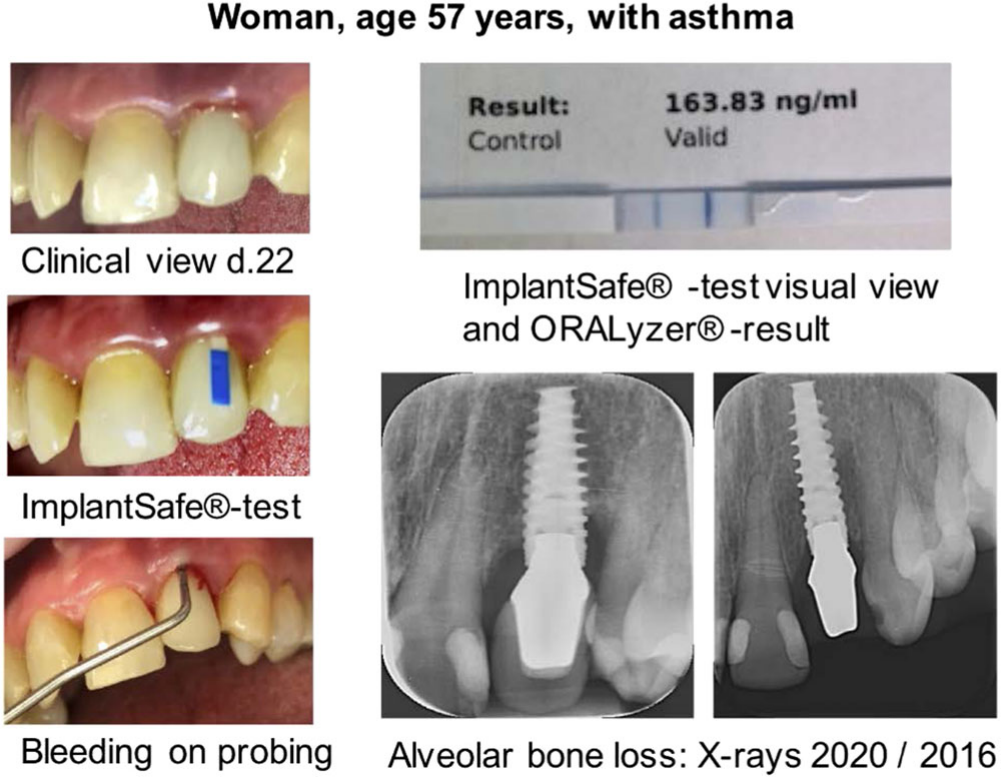
An example of an upper jaw dental implant with a clinical view, X‐rays showing advanced horizontal alveolar bone destruction, and active matrix metalloproteinase‐8 point‐of‐care/chairside enzyme test assay showing a significantly elevated, strong positive (++) test result

Figure 2 demonstrates an example of an upper jaw dental implant. A deepened peri‐implant pocket and BOP were observed on clinical evaluation. X‐rays showed advanced horizontal alveolar bone destruction. The aMMP‐8‐PoC test assay was a strong positive (163.83 ng/ml).

### 3.3 Diagnostic accuracy of the studied biomarkers

**Figure 3:**
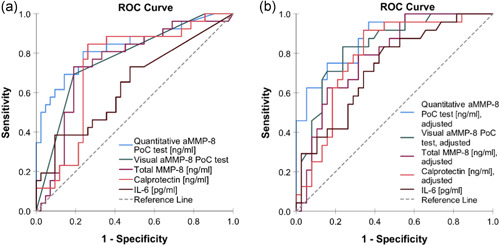
Receiver operating characteristic (ROC) analysis illustrating the diagnostic ability of the biomarker candidates to discriminate healthy implant from peri‐implantitis: (a) unadjusted (*n* = 68) and (b) adjusted for gender, age of dental implant and smoking (*n* = 62)

ROC analysis for the studied biomarkers and their discriminatory ability to classify peri‐implantitis and health is presented in Table 3 and Figure 3. Both univariable and adjusted (gender, age of dental implant, and smoking) models were assessed. ROC curves showed the highest diagnostic performance for quantitative and visual aMMP‐8‐PoC tests both in univariable (AUC = 0.833 and 0.773, respectively) and adjusted models (AUC = 0.880 and 0.883, respectively). They were followed by total MMP‐8 (AUC = 0.750 for univariable model and AUC = 0.788 for adjusted model), calprotectin (AUC = 0.736 and 0.787) and IL‐6 (AUC = 0.637 and 0.726).

**Table 3:** Diagnostic potential of biomarkers to classify peri‐implant health and peri‐implantitis

Biomarker/Univariable model (*n* = 68)	AUC (95% CI)	*p* value	Cut‐off point	Se (%)	Sp (%)	FN (%)	FP (%)	Acc (%)	MCC
Quantitative aMMP‐8 PoC test (ng/ml)	0.833 (0.728–0.938)	<.001	63.1	80.8	76.2	13.5	32.3	77.9	0.556
Visual aMMP‐8 PoC test^a^	0.773 (0.657–0.888)	<.001	1.5	69.2	81.0	19.0	30.8	76.5	0.502
Total MMP‐8 (ng/ml)	0.750 (0.627–0.872)	.001	2.68	73.1	81.0	17.1	29.6	77.9	0.537
Calprotectin (ng/ml)	0.736 (0.611–0.861)	.001	4772.0	84.6	73.8	11.4	33.3	77.9	0.568
IL‐6 (pg/ml)	0.637 (0.498–0.776)	.059	1.46	38.5	90.5	29.6	28.6	70.6	0.348

**Biomarker/Adjusted model** b **(*n*** = **62)**	**AUC (95% CI)**	***p *** **value**	**Cut‐off point** c	**Se (%)**	**Sp (%)**	**FN (%)**	**FP (%)**	**Acc (%)**	**MCC**
Quantitative aMMP‐8 PoC test (ng/ml)	0.880 (0.798–0.963)	<.001	0.363	75.0	84.2	15.8	25.0	80.6	0.592
Visual aMMP‐8 PoC test^a^	0.833 (0.731–0.935)	<.001	0.333	83.3	76.3	12.1	31.0	79.0	0.582
Total MMP‐8 (ng/ml)	0.788 (0.677–0.900)	<.001	0.321	79.2	68.4	16.1	38.7	72.6	0.464
Calprotectin (ng/ml)	0.787 (0.672–0.903)	<.001	0.300	91.7	65.8	7.4	37.1	75.8	0.564
IL‐6 (pg/ml)	0.726 (0.600–0.853)	.003	0.300	83.3	55.3	16.0	45.9	66.1	0.383

*Note*: The Youden index was used to define the optimal cut‐offs for each biomarker from the ROC curves.

Abbreviations: Acc, accuracy; AUC, area under the ROC curve; CI, confidence interval; FN, false negatives; FP, false positives; MCC, Matthews correlation coefficient; PoC, point‐of‐care; Se, sensitivity; Sp, specificity.

^a^ Visual aMMP‐8 PoC test (0, negative; 1, weak positive; 2, strong positive).

^b^ Adjusted for gender, age of dental implant and smoking.

^c^ Cut‐off points are the optimal predicted probabilities for the adjusted logistic regression model.

Optimal cut‐off points to classify healthy and peri‐implantitis implants were defined for each biomarker (unadjusted and adjusted for gender, age of dental implant and smoking) by using Youden's index (Table 3). The best accuracy (unadjusted model) was obtained by calprotectin (accuracy = 77.9% and MCC = 0.57), quantitative aMMP‐8‐PoC test (accuracy = 77.9% and MCC = 0.56) and total MMP‐8 (accuracy = 77.9% and MCC = 0.54). However, in adjusted models, the best accuracy was observed for quantitative aMMP‐8‐PoC test (accuracy = 80.6% and MCC = 0.59), visual aMMP‐8‐PoC test (accuracy = 79.0% and MCC = 0.58) and calprotectin (accuracy = 75.8% and MCC = 0.56).

### 3.4 MMP‐8, MMP‐2, and MMP‐9 species versus peri‐implant health and peri‐implantitis

**Figure 4:**
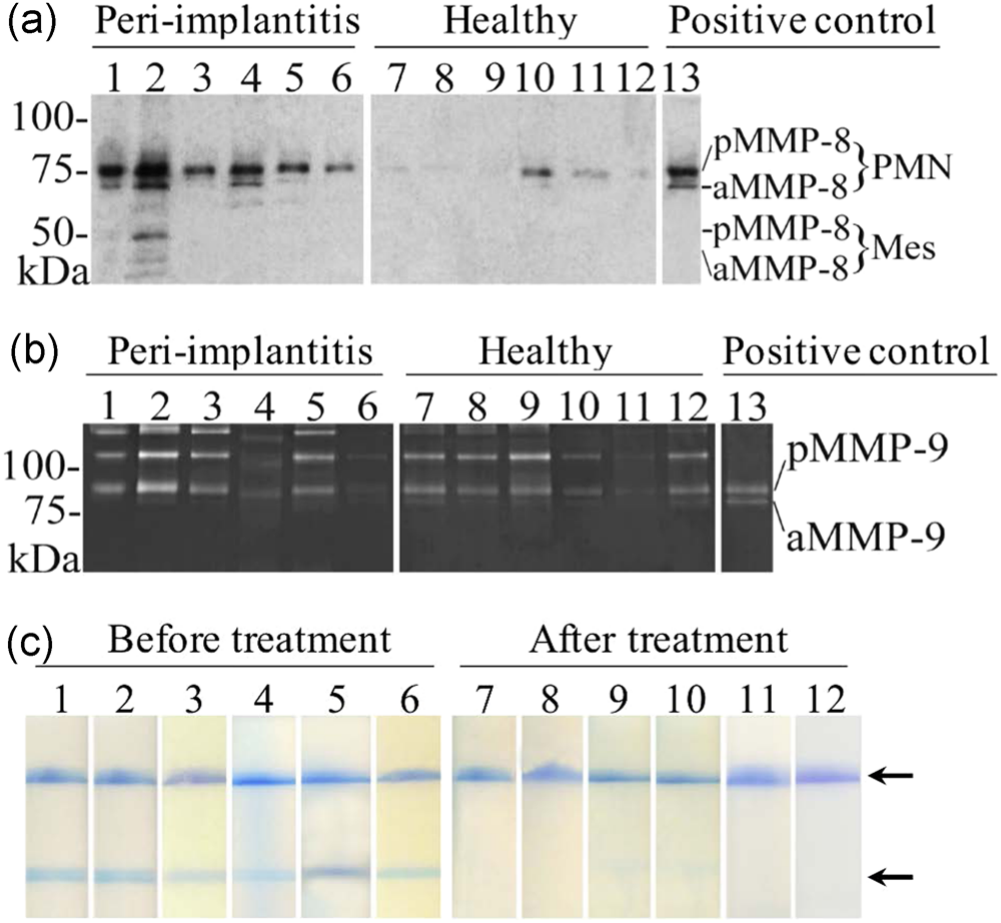
(a) Western immunoblot analysis of peri‐implantitis (Lanes 1–6) and healthy (Lanes 7–12) peri‐implant sulcular fluid (PISF) for matrix metalloproteinase (MMP)‐8; Lane 13 represents a positive control of neutrophil MMP‐8. Here, pMMP‐8 and aMMP‐8 indicate pro‐ and active species, respectively, in corresponding neutrophil (PMN)‐type and mesenchymal (–Mes) fibroblast‐type MMP‐8 isoforms. Observe activation and fragmentation of MMP‐8 in peri‐implantitis PISF versus healthy PISF. (b) Gelatin‐zymographic analysis of peri‐implantitis (Lanes 1–6) and healthy (Lanes 7–12) PISF; Lane 13 represents positive control of neutrophil gelatinase B. Observe similar forms and levels of MMP‐9 but hardly not at all MMP‐2 (in the 62–72 kDa areas) in both peri‐implantitis and healthy PISF. Here, pMMP‐9 and aMMP‐9 indicate pro‐ and active species, respectively. Mobilities of the molecular weight markers are indicated on the left (Panels a and b). (c) The effect of treatments of peri‐implantitis on aMMP‐8 PoC/chair‐side PISF visual test outcomes (+ and −, *n* = 6) before (Lanes 1–6) and 4–6 weeks after (Lanes 7–12) treatments. Two lines indicate a positive test (+, >20 ng/ml aMMP‐8 in the PISF) and one line indicates a negative test (−, <20 ng/ml aMMP‐8 in the PISF) as pointed by the arrows on the right

Western immunoblot analysis with independent polyclonal and specific MMP‐8 antibody revealed that while consistently elevated MMP‐8 species in activated and fragmented forms could be detected in the diseased PISF (Figure 4a, Lanes 1–6), MMP‐8 was either hardly detectable or detectable in latent form only in the healthy PISF (Figure 4a, Lanes 7–12). Gelatin zymographic analysis of MMP‐2 and MMP‐9 using the same PISF samples could not differentiate between peri‐implant health and disease (Figure 4b, Lanes 1–12).

Finally, Figure 4c demonstrates the successful effect of treatment of peri‐implantitis according to the Swedish National Guidelines. (https://www.socialstyrelsen.se/globalassets/sharepoint-dokument/artikelkatalog/nationella-riktlinjer/2011-5-1.pdf), monitored by aMMP‐8‐PoC test, evidencing changes from positive, ≥20 ng/ml (elevated risk), to negative, <20 ng/ml (no/low risk), of the visually recorded real‐time PoC test outcomes.

## 4 DISCUSSION

In this study, we observed a significant association between the aMMP‐8‐PoC/chairside enzyme test results and the prevalence of peri‐implantitis and clinical peri‐implant risk factors. We found that peri‐implant tissue damage involving active collagenolysis, indicated by the elevated aMMP‐8 levels, was significantly more common in dental implants that had peri‐implantitis compared with healthy implants. Furthermore, low aMMP‐8 levels (<20 ng/ml) in PISF were clearly linked to healthy implant defined as the absence of the combination of X‐ray findings (bone loss > 2 mm), BOP, and peri‐implant pockets of ≥3 mm around the dental implant. On the other hand, the increase in aMMP‐8 levels was associated with the combination of these three clinical measurements and parameters (peri‐implantitis): moderate aMMP‐8 levels (≥20 ng/ml) indicated the elevated risk of peri‐implantitis and high aMMP‐8 levels (≥80 ng/ml) indicated an even higher risk for the three clinical peri‐implantitis parameters that may suggest a more rapid disease progression. This was also confirmed with western immunoblot analysis utilizing an independent polyclonal and specific MMP‐8 antibody demonstrating the elevation of activated and fragmented MMP‐8 species in the diseased PISF, but not in the healthy PISF that contained only latent MMP‐8 species. These findings support and further extend previous studies that have shown not only the direct role of collagenase activity (active MMP‐8, not latent/total MMP‐8) in the progression of attachment loss, (Kiili et al., 2002; Kivelä‐Rajamäki et al., 2003; Lee et al., 1995; Mancini et al., 1999; Romanelli et al., 1999; Sorsa et al., 2006) but also aMMP‐8 levels in oral fluids correlating well with clinical periodontal/peri‐implant parameters in adults and adolescents (Heikkinen et al., 2019; Izadi Borujeni et al., 2015; Lähteenmäki et al., 2020; Lee et al., 1995; Leppilahti et al., 2014; Lorenz et al., 2017; Räisänen et al., 2019; Räisänen et al., 2020; Schmalz et al., 2019; Sorsa, Alassiri, et al., 2020; Sorsa, Bacigalupo, et al., 2020; Xu et al., 2008). Furthermore, previous studies indicate that microbial burden could act as an up‐regulator in the aMMP‐8 cascade (proteolytic activation of pro‐MMP‐8 to aMMP‐8) (Gürsoy et al., 2018; Nieminen et al., 2018; Sorsa et al., 1992, 1995). The onset of disease is, hence, heralded by the elevation of periodontal/peri‐implant tissue destruction associated biomarkers, indicating collagenolysis.

We extended our analysis to compare the aMMP‐8‐PoC test assay measuring aMMP‐8 and its levels with other potential biomarkers (total MMP‐8, calprotectin, and IL‐6 ELISAs). Although their levels, in addition to aMMP‐8, were elevated in peri‐implantitis compared with healthy implants, aMMP‐8 had the best diagnostic accuracy to discriminate peri‐implant health and disease as judged from ROC‐analysis. This supports and further extends our prior studies that have shown that aMMP‐8 measured by the aMMP‐8‐PoC test assay is seemingly superior to other tested potential biomarkers, including neutrophil elastase, calprotectin, tissue inhibitor of MMPs (TIMP)‐1, myeloperoxidase, BOP, and MMP‐9 in classifying dental implants as healthy or diseased (Golub et al., 2020; Lähteenmäki et al., 2020; Sorsa, Bacigalupo, et al., 2020). Moreover, we found that successful peri‐implantitis treatment converted the elevated (≥20 ng/ml) aMMP‐8 levels in the PISF samples to low/healthy (<20 ng/ml) aMMP‐8 levels in PISF. Thus, our results support previous studies that have shown that the aMMP‐8 levels can be used to monitor and assess periodontal/peri‐implant treatment success and failure (Alassiri et al., 2018; Golub et al., 2020; Lähteenmäki et al., 2020; Leppilahti et al., 2014, 2015; Sorsa, Bacigalupo, et al., 2020; Thierbach et al., 2016) However, the definition of healthy dental implant and peri‐implantitis has eventually an impact on the balance between sensitivity and specificity and should be considered when comparing the results in this study with other studies. There is quite a variation in the case definitions of peri‐implantitis in the literature, yet the main criteria for peri‐implantitis in most of the studies have been based on BOP, PD** **≥ 3 mm, and cases of crestal bone loss of ≥2 mm (Natto et al., 2019; Renvert et al., 2018). And in peri‐implant health, PD should in general be ≤5 mm (Renvert et al., 2018). Thus, the definition used in this study is in line with what has been considered peri‐implantitis in the literature. The rational and the reason for more strict definition regarding PD in this study was to make sure that the dental implants that were defined healthy (in terms of clinical signs) would really be healthy.

To ensure long‐term periodontal/peri‐implant health, it is imperative to maintain collagen stability and integrity. As discussed earlier, traditional tools like dental probes, X‐rays, or clinical examination by inspection are unable to reliably detect active collagenolysis or the pivotal point between health and disease. Analyzing oral fluids for aMMP‐8 levels offers a noninvasive and nonbacteremic method to make the invisible process of collagenolysis visible (Gul et al., 2020). Based on the result of the aMMP‐8 analysis, it is possible to evaluate the current state and disease activity of the periodontal/peri‐implant tissues and thus conveniently assess the risk of both developing and ongoing collagen degradation/collagenolysis, to predict possible future attachment tissue loss (Gul et al., 2020; Lee et al., 1995; Leppilahti et al., 2014, 2015). In other words, the beauty of the aMMP‐8 POCT for clinicians is its ability to alarm collagenolytic tissue destruction prior appearance of clinical manifestations, that is, it makes invisible visible. This could help clinicians to personalize more precisely secondary prevention protocols based on breakdown intensity (collagenolytic activity) and, at the same time, improve patient compliance in terms of oral hygiene maintenance and adherence to recall appointments. At present, most implant patients are called in once a year, which may not be sufficient to capture disease initiation. Limitations of the study include the small sample size and lack of longitudinal follow‐up data to confirm the ongoing progressing attachment loss. The clinical measurements and parameters used in this study to define peri‐implantitis are not direct indicators of active progressing attachment loss, although they are associated with the disease. This may, for example, partly explain the number of false positives, as previous studies indicate that the active form of MMP‐8 is an important biomarker for progressing periodontal and peri‐implant attachment loss (Heikkinen et al., 2019; Izadi Borujeni et al., 2015; Kiili et al., 2002; Kivelä‐Rajamäki et al., 2003; Lähteenmäki et al., 2020; Lee et al., 1995; Leppilahti et al., 2014; Lorenz et al., 2017; Mancini et al., 1999; Räisänen et al., 2019, 2020; Romanelli et al., 1999; Schmalz et al., 2019; Sorsa, Alassiri, et al., 2020; Sorsa, Bacigalupo, et al., 2020; Sorsa et al., 2006; Xu et al., 2008). Currently, there is no gold standard for disease activity and real‐time progression of the disease. Thus, biomarker studies, like the present one, are forced to compare the measurements against the current clinical diagnostic parameters even if not necessarily perfectly accurate.

The 2018 classification system of periodontitis has the necessary framework to implement biomarkers (Tonetti et al., 2018). Similarly, biomarkers could be considered to be integrated for the assessment of peri‐implant in the official classification system of peri‐implant diseases (Berglundh et al., 2018). Recent studies regarding periodontitis as described by the new classification system of periodontitis have presented promising results of the use of aMMP‐8 as an adjunctive diagnostic tool to traditional clinical methods (Chaparro et al., 2020; Keles Yucel et al., 2020; Öztürk et al., 2021; Sorsa, Alassiri, et al., 2020). Furthermore, the present study and our previous studies indicate similarly that aMMP‐8 is a potential candidate to act as a biomarker in the disease classification for peri‐implantitis (Alassiri et al., 2018; Golub et al., 2020; Lähteenmäki et al., 2020; Sorsa, Bacigalupo, et al., 2020; Thierbach et al., 2016). In addition to these diseases, elevated aMMP‐8 concentrations in oral fluids are associated with (pre)diabetes and gestational diabetes, and the aMMP‐8‐PoC enzyme test can also identify the risk of diabetes (and eventually other serious systemic diseases) as well as the destructive oral side‐effects of radiotherapy for head and neck cancer (Chaparro et al., 2020; Grigoriadis et al., 2019, 2021; Keskin et al., 2020; Räisänen et al., 2020). Overall, aMMP‐8 has the potential to influence and improve not only the diagnostics of periodontal and peri‐implant diseases but also the interdisciplinary collaboration between dental and healthcare professionals in their pursuit of attaining good oral and general health for their patients (Räisänen et al., 2020).

## 5 CONCLUSION

aMMP‐8‐PoC/chairside test can be conveniently implemented to alert for and detect active collagenolysis affecting peri‐implant tissues, both in the early and advanced stages of the disease. Active and fragmented MMP‐8 exhibits a strong and significant association with peri‐implantitis as compared to total MMP‐8 and other biomarkers. The data demonstrate that an aMMP‐8 chairside assay can be used as a convenient and reliable adjunctive tool in the diagnosis and monitoring of peri‐implantitis. These results need to be clarified in further studies.

## CONFLICT OF INTERESTS

Prof. Timo Sorsa is the inventor of US 5,652,223, 5,736,341, 5,864,632, 6,143,476 and US 2017/0023571A1 (issued June 6, 2019), WO 2018/060553 A1 (issued May 31, 2018), 10,488,415 B2, and US 2017/0023671A1 and Japanese Patent 2016‐554676. Dr. Pirjo Pärnänen has a patent EP 2585087B1. Dirk‐Rolf Gieselmann is the inventor of US 20170023671A1 patent. Other authors report no conflicts of interest related to this study. The funders had no role in the design of the study; in the collection, analyses, or interpretation of data; in the writing of the manuscript, or in the decision to publish the results.

## ETHICS STATEMENT

The study was conducted according to the guidelines of the Declaration of Helsinki and approved by the Ethics Committee of the Helsinki University Hospital, Finland (106§/26.06.2019; dnro HUS/1271/2019) and Regionala etikprövingsnämnden i Stockholm, (EPN) (2016‐08‐24/2016/1:8 and 2016‐1‐24; Dnr 2016/1410‐31/1). Informed consent was obtained from all subjects involved in the study.

## AUTHOR CONTRIBUTIONS

*Collected the samples and did the clinical diagnosis, patient treatment*: Hanna Lähteenmäki. *Directed the lab work on biomarkers*: Taina Tervahartiala. *Statistical analysis, writing*: Ismo T. Räisänen. *Laboratory work of samples, writing, clinical diagnosis, patient treatment*: Pirjo Pärnänen. *Patient clinical data evaluation, medical specialist*: Matti Mauramo. *Writing the paper, clinical planning, English language*: Shipra Gupta. *Writing paper, English language, clinical data evaluation*: Victoria Sampson. *Writing paper, revision planning and writing*: Nilminie Rathnayake. *Patient treatment, clinical data evaluation, and writing*: Anna‐Maria Heikkinen. *Laboratory work/biomarker analysis of samples, writing the paper*: Saeed Alassiri. *Development of aMMP‐8 poct test, writing of the paper*: Dirk‐Rolf Gieselmann. *Writing of paper and clinical data evaluation*: Roland Frankenberger. Discovery and development of the aMMP‐8: *Timo Sorsa*.

## Data Availability

The data that support the findings of this study are available on reasonable request from the corresponding author. The data are not publicly available due to privacy and ethical restrictions.
